# Identification of myocardial diffuse fibrosis by 11 heartbeat MOLLI *T*_1_ mapping: averaging to improve precision and correlation with collagen volume fraction

**DOI:** 10.1007/s10334-017-0630-3

**Published:** 2017-06-12

**Authors:** Vassilios S. Vassiliou, Katharina Wassilew, Donnie Cameron, Ee Ling Heng, Evangelia Nyktari, George Asimakopoulos, Anthony de Souza, Shivraman Giri, Iain Pierce, Andrew Jabbour, David Firmin, Michael Frenneaux, Peter Gatehouse, Dudley J. Pennell, Sanjay K. Prasad

**Affiliations:** 1 Norwich Medical School, University of East Anglia, Bob Champion Research and Education Building, Norwich Research Park, Norwich, NR4 7UQ UK; 2 CMR Unit and NIHR Cardiovascular Biomedical Research Unit, Royal Brompton Hospital, Sydney Street, London, SW3 6NP UK; 3 Imperial College, National Heart and Lung Institute, London, UK; 4 The Pathology Department, Rigshospitalet, University Hospital of Copenhagen, Blegdamsvej 9, 2100 Copenhagen, Denmark; 5 Siemens Medical Solutions USA, Inc, Chicago, USA; 6 Department of Cardiology, St Vincent’s Hospital, Darlinghurst, Australia

**Keywords:** *T*_1_ mapping, MOLLI, Correlation with collagen volume fraction, Precision, Extracellular volume, Gadolinium

## Abstract

**Objectives:**

Our objectives involved identifying whether repeated averaging in basal and mid left ventricular myocardial levels improves precision and correlation with collagen volume fraction for 11 heartbeat MOLLI *T*_1_ mapping versus assessment at a single ventricular level.

**Materials and methods:**

For assessment of *T*_1_ mapping precision, a cohort of 15 healthy volunteers underwent two CMR scans on separate days using an 11 heartbeat MOLLI with a 5(3)3 beat scheme to measure native *T*_1_ and a 4(1)3(1)2 beat post-contrast scheme to measure post-contrast *T*_1_, allowing calculation of partition coefficient and ECV. To assess correlation of *T*_1_ mapping with collagen volume fraction, a separate cohort of ten aortic stenosis patients scheduled to undergo surgery underwent one CMR scan with this 11 heartbeat MOLLI scheme, followed by intraoperative tru-cut myocardial biopsy. Six models of myocardial diffuse fibrosis assessment were established with incremental inclusion of imaging by averaging of the basal and mid-myocardial left ventricular levels, and each model was assessed for precision and correlation with collagen volume fraction.

**Results:**

A model using 11 heart beat MOLLI imaging of two basal and two mid ventricular level averaged *T*_1_ maps provided improved precision (Intraclass correlation 0.93 vs 0.84) and correlation with histology (*R* ^2^ = 0.83 vs 0.36) for diffuse fibrosis compared to a single mid-ventricular level alone. ECV was more precise and correlated better than native *T*_1_ mapping.

**Conclusion:**

*T*_1_ mapping sequences with repeated averaging could be considered for applications of 11 heartbeat MOLLI, especially when small changes in native *T*_1_/ECV might affect clinical management.

## Introduction

The longitudinal relaxation time, *T*_1_, of the myocardium is regarded as a useful imaging biomarker, as it can change with cardiac pathology and is known to be associated with functional capacity and mortality [1–5]. Traditionally, late gadolinium enhancement (LGE) cardiovascular magnetic resonance (CMR) exploits changes in *T*_1_ following administration of a gadolinium–based contrast agent (Gd): namely, shortening of *T*_1_, which manifests as bright signal intensity on conventional inversion-recovery gradient echo sequences. This has been used as the primary tool for identification of focal (or replacement) fibrosis, indicative of scar, and has entered clinical routine for multiple pathologies, including myocardial infarction and viability [6], cardiomyopathy [2, 7], congenital heart disease [8] and valvular heart disease [5, 9].

A limitation of *T*_1_-weighted inversion recovery sequences is that they rely on the nulling of signal in normal myocardium to highlight concentrations of Gd in fibrotic areas. In cases of diffuse (interstitial) fibrosis, however, where the myocardium can be globally affected, the myocardial signal may appear isointense and, hence, lack sensitivity in identifying fibrosis [10]. To address this unmet clinical need and allow identification of diffuse myocardial fibrosis, new *T*_1_ mapping CMR sequences have been developed, based on the Modified Look-Locker inversion recovery (MOLLI) sequence first described by Messroghli and colleagues [11]. These allow imaging of extracellular volume fractions as a surrogate for diffuse fibrosis [12–16].

Recent iterations of the MOLLI *T*_1_ mapping sequence acquire a total of 8 or 9 *T*_1_-weighted images over 11 heart beats [14, 17], in a 5b(3b)3b scheme pre-contrast, and a 4b(1b)3b(1b)2b scheme post-contrast, where ‘b’ denotes beats and the values in brackets indicate pause intervals. These shortened acquisitions enable rapid breath-held imaging in around 8–12 s, depending on heart-rate, compared to the 15–20 s required by older MOLLI sequences, which typically acquired *T*_1_-weighted images in a 3b(3b)3b(3b)5b scheme [11, 18]. While reducing the number of *T*_1_-weighted MOLLI source images from 11 to 8/9 may reduce *T*_1_ precision, it also has the distinct advantage of increased reliability through better patient breath-holding. Therefore, the newer, faster sequences have enabled extended applications in patients with poorer breath-holding ability. However, before a new sequence is fit for clinical application, assessment of precision and accuracy is required, benchmarked against the reference standard of histologically derived quantification of diffuse fibrosis.

We examined an 11 heartbeat (11HB) MOLLI prototype, by Siemens, with native 5b(3b)3b and post-contrast 4b(1b)3b(1b)2b, which is referred to in the main text as MOLLI for brevity. This sequence has undergone little exploratory validation, and thus the aim of this work is to:

1. explore the effect of six imaging models (models A–F, Table 1) on precision and correlation with collagen volume fraction (CVF), which we use as a surrogate for accuracy; these models use incremental averaging of left ventricular slices (up to two averages at basal and up to two at mid-ventricular levels, before and after Gd) here referred to as “incremental inclusion of images”, and will be compared with the conventional use of only one mid-ventricular level slice;

**Table 1:** Using the four native and four post-Gd images it was possible to construct a total of 6 *T*_1_ mapping models

	Basal level	Repeated	Mid-level	Repeated
Model A			x	
Model B	x			
Model C			x	x
Model D	x	x		
Model E	x		x	
Model F	x	x	x	x

The “x” marks the slice location of inclusion of the additional imaging
2. histologically validate the 5b(3b)3b and 4b(1b)3b(1b)2b 11 heart beat MOLLI prototype in patients who have left ventricular myocardial biopsy at the time of aortic valve surgery; and
3. explore the relative precision and correlation with CVF of native *T*_1_ mapping, partition coefficient, and extracellular volume fraction (ECV).

## Materials and methods

### Population cohorts

Two cohorts of participants were recruited for this work: a group of healthy volunteers for estimating *T*_1_ mapping precision, and a group of aortic stenosis patients for estimating *T*_1_ mapping correlation with CVF. Written consent was obtained from all patients and volunteers, and the study was conducted after local research ethics approval from the Royal Brompton and Harefield NHS Foundation trust and in accordance with the Declaration of Helsinki principles for Medical Research.

### Cohort for estimating reproducibility/precision

Healthy volunteers taking no medication and with no known medical conditions were recruited following advertisements on public notice boards. The volunteers underwent a detailed health questionnaire and blood screening to measure haemoglobin, renal function, liver function, thyroid function, and C-reactive protein. The bloods were measured in a clinical biochemistry laboratory. Additionally, blood pressure, heart rate, and temperature were recorded. All volunteers underwent electrocardiography. In the event that an abnormality (e.g., hypertension, ECG abnormalities, renal failure) was detected that could have affected the results, the participant was excluded.

### Cohort for estimating correlation with collagen volume fraction

A cohort of patients with severe aortic stenosis, scheduled for surgical aortic valve replacement, was recruited from outpatient clinics or inpatient wards at the Royal Brompton Hospital, London, UK. The patients were excluded if they had any contraindications to CMR or if they were scheduled for surgery for more than one valve. Patients who were scheduled for concurrent aortic valve replacement and coronary artery by-pass grafting (CABG) were included. As this was a correlation study, the patients were not excluded if they had treated hypertension, hyperlipidaemia, or diabetes.

Assuming similar correlations to histological CVF to previous published data [12], we estimated that a minimum of five patients would be required to detect an *R* ^2^ correlation of at least 0.8 with 80% power. We opted to double this number to ten patients, giving us 90% power to detect a statistically significant correlation assuming that the true *R* ^2^ correlation was at least 0.6.

### Cardiovascular magnetic resonance

All CMR imaging was performed on a 1.5 T scanner (Siemens Avanto, Erlangen, Germany). The quadrature body coil was used for radiofrequency transmission with 12–18 elements of an anterior and posterior cardiac parallel array coil for reception. The standard manufacturer wireless-ECG was used as in routine clinical work.

#### *T*_1_ mapping acquisition

The *T*_1_ mapping acquisition protocol was the same for both volunteers and aortic stenosis patients. Following adjustment of the scanner reference frequency to the predominant Larmor nuclear magnetic resonance precession frequency of the signal received from a cuboid volume region over the left-heart (“volume adjust”, ideally performed end-expiration), MOLLI *T*_1_ mapping was applied with the following parameters: Single-shot balanced steady-state free-precession images, field-of-view = 360 × 306 mm, slice thickness = 8 mm, alpha pulse flip angle = 35^o^, 7/8ths partial *k*_y_ acquisition with 1/8th zero-filling, a generalised autocalibrating partially parallel acquisition acceleration factor of 2 with 24 central *k*_y_ lines fully acquired to obtain receiver coil profiles, and 1085 Hz/pixel ADC sampling bandwidth. The *T*_1_ weighting was initially TI = 120 ms, incrementing by 80 ms at the start of each Look-Locker set. Two different MOLLI acquisitions were used depending on participant heart rates: a high-resolution acquisition for heart rates less than 90 bpm and a low-resolution acquisition for heart rates greater than 90 bpm. Two different sequences were required because the high-resolution acquisitions were too long to fit into the stationary diastole phase for faster heart rates. The following parameters varied between high-resolution and low-resolution setups, and these differences have been shown to have negligible impact on *T*_1_ values while maximising image quality [10].

High resolution: TR/TE = 2.6/1.12 ms, 84 acquired PE lines, single-shot image duration 220 ms, Acquired pixel size 1.4 mm (FE) × 2.1 mm (PE).

Low resolution: TR/TE = 2.4/1.0 ms, 76 acquired PE lines, single-shot image duration 182 ms, Acquired pixel size 1.9 mm (FE) × 2.4 mm (PE).

Further, for the low- and high-resolution MOLLI acquisitions, two different MOLLI sampling schemes were used pre- and post-contrast [10]: a 5b(3b)3b scheme was used for native *T*_1_ values and a 4b(1b)3b(1b)2b scheme was used for post-contrast *T*_1_.

A total of four native *T*_1_ maps were acquired (one basal and repeated once; one mid-ventricular level and repeated once). Then, starting fifteen minutes after Gd administration, post-Gd MOLLI *T*_1_ maps were acquired at basal and mid-ventricular levels (and repeated once at each level, giving rise to four post-Gd *T*_1_ maps) using the 4b(1b)3b(1b)2b scheme, again with low or high resolution depending on the heart rate. In total, four native *T*_1_ maps and four post-contrast *T*_1_ maps were obtained. The addition of a complete *T*_1_ mapping protocol to a clinical scan increased the total scan time by about 5–7 min per patient.

To assess interscan reproducibility, the healthy volunteers underwent a second CMR scan within 60 days of the first. To mimic routine clinical practice, the staff undertaking the second scan were unaware of the exact slice position used for acquisition in the first scan. The aortic stenosis patients underwent a single CMR scan as close to the day of surgery as possible.

### Image post-processing and analysis

**Fig. 1:**
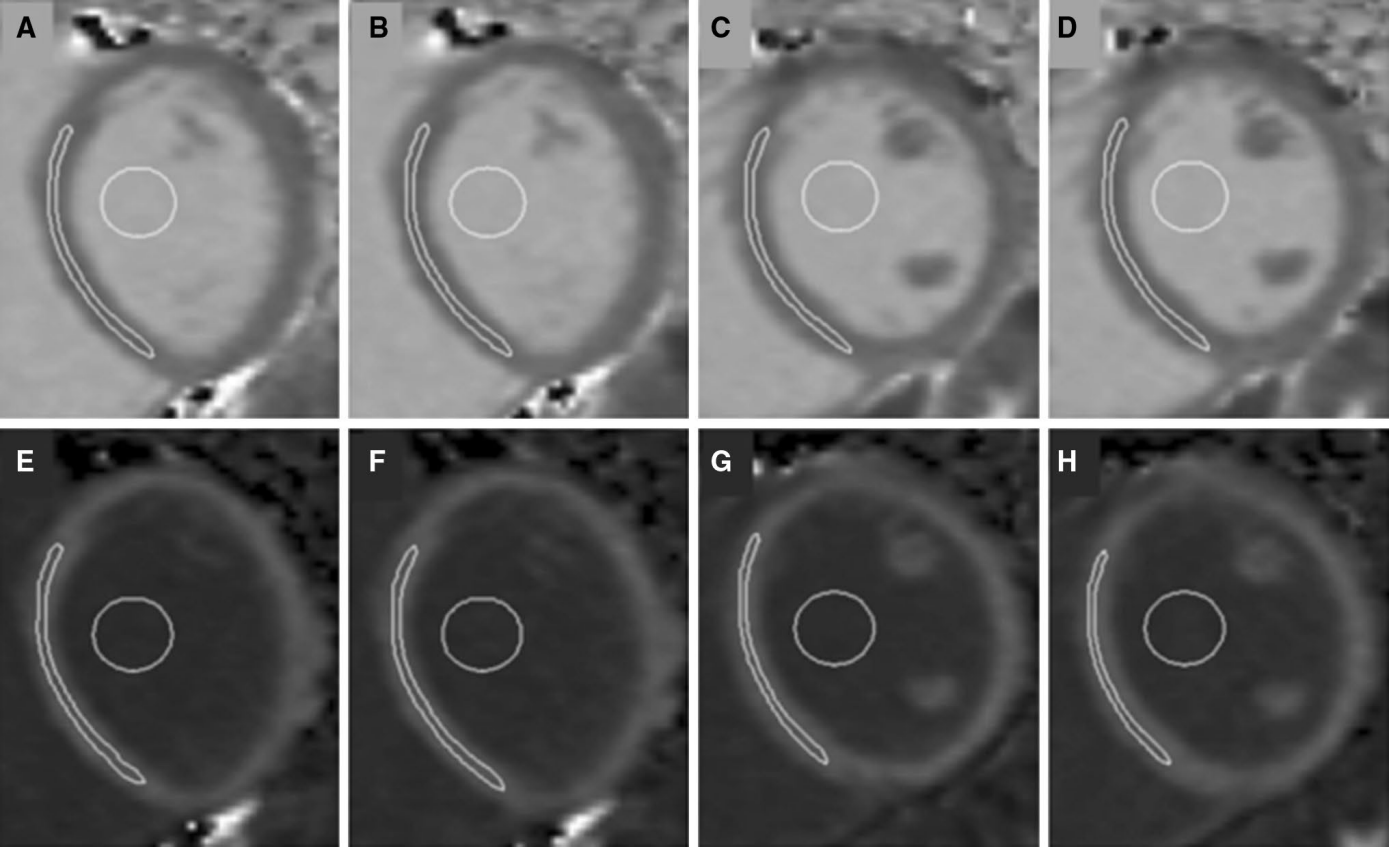
Representative short axis images from a healthy volunteer with native *T*_1_ maps (**a**–**d**) and post gad *T*_1_ maps (**e**–**h**). One basal and one mid-ventricular level were selected and imaging at each level was repeated both before and after the administration of Gd (**a**, **b** native *T*_1_ basal; **e**, **f** post-Gd basal; **c**, **d** native *T*_1_ mid-ventricular level, **g**, **h**, mid-ventricular level post-Gd). Regions of interest (ROI) were drawn in the myocardium and blood

**Fig. 2:**
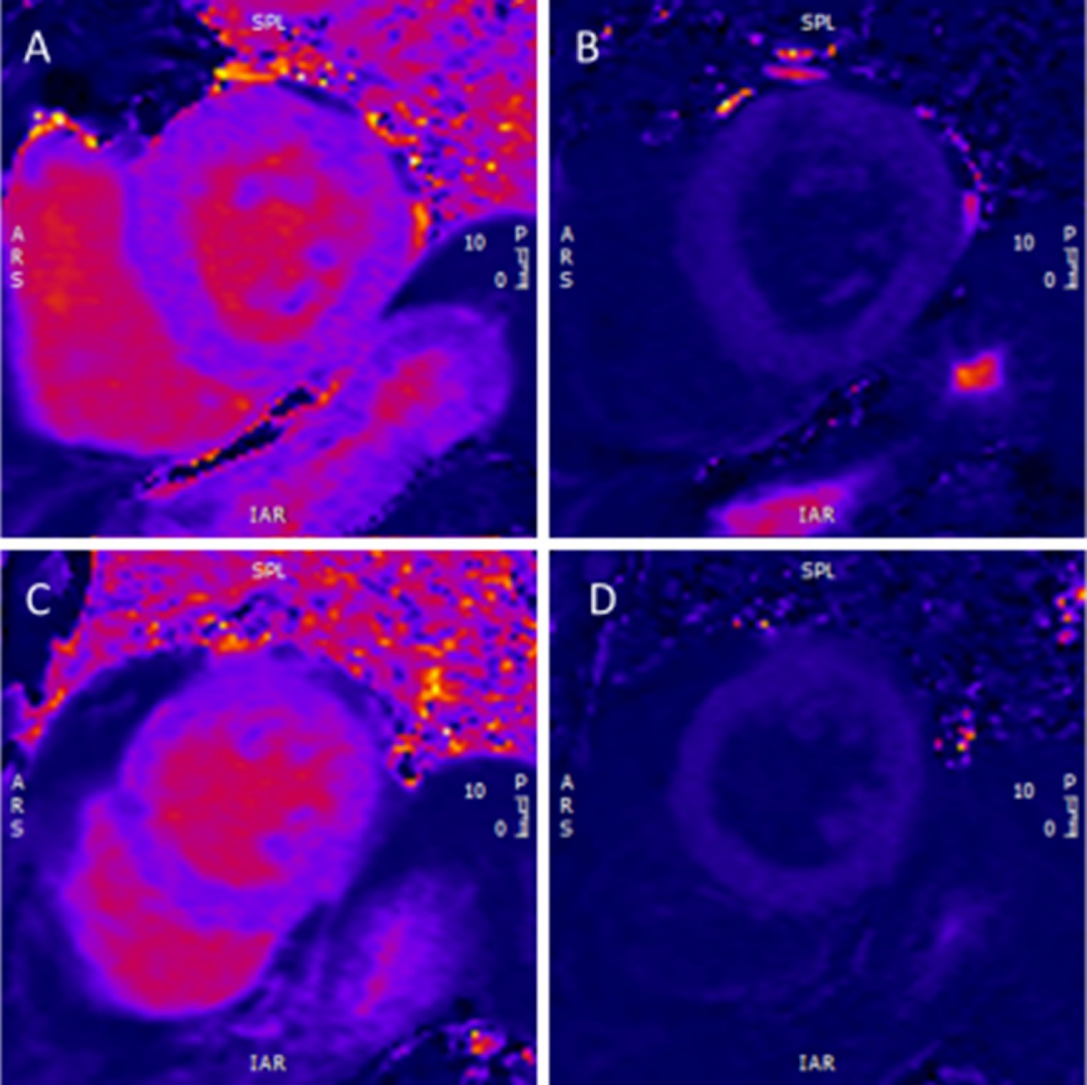
**a** showing a basal slice with native *T*_1_ mapping using the high resolution 5(3)3 beat sequence as the heart rate was 58 bpm. **b** shows the post-Gd image using a high resolution 4(1)3(1)2 beat sequence. **c** shows the native *T*_1_ mapping and **d** the post-Gd contrast *T*_1_ mapping using the same high resolution sequence as in (**a**, **b**) respectively, but at the mid-ventricular level

For quantification of LV function, volumes, and *T*_1_ values, dedicated software CMR Tools (Cardiovascular Imaging Solutions, London, UK, Fig. 1) was used by experienced (level 3 SCMR) blinded operators. Visual inspection of the colour maps was undertaken both in-line on the Siemens platform and off-line using CMR 42 (Circle CVI, Calgary, Canada, Fig. 2).

MOLLI *T*_1_ maps were generated automatically in-line on the scanner workstation, following motion correction and pixel-by-pixel curve fitting with Look-Locker correction. DICOM images were anonymised and maps were analysed in a blinded fashion by two observers to evaluate interstudy and interobserver variability. A third observer adjudicated values of >5% relative difference. Native and post-Gd MOLLI source images were inspected and a quality score from 1 to 5, with five being excellent and 1 very poor, was recorded for respiratory drift, cardiac motion artefact, and appropriate cardiac triggering of each image, before proceeding with measurements on the derived *T*_1_-maps. Only patients scoring ≥4 in all categories were subsequently included in the analysis.

Regions of interest (ROIs) were drawn on the *T*_1_ maps (Fig. 1) within the mid-wall of the septum, with care being taken to avoid partial-volume or cardiac-motion-related blurring of the myocardium-blood boundary, where blood signal contaminates myocardial data. A second set of ROIs were drawn in the blood, avoiding myocardium and papillary muscles. A standardised approach was undertaken in all patients with regards to the location of the ROI, and all areas were included, even if they were subsequently shown to have LGE. This had been decided a priori, as in patients where only native *T*_1_ mapping is undertaken, one does not have the subsequent benefit of knowing where LGE is present, and therefore our protocol was standardised in this way for all the patients.

Once native and post-Gd myocardial and blood values and haematocrit were obtained, extracellular volumes (ECVs) were calculated as shown in Eq. (1):

1 $${\text{ECV}} = \left( {1 - {\text{haematocrit}}} \right)* \frac{{\left( {\frac{1}{{T_{1} myo post}} - \frac{1}{{T_{1} myo pre}}} \right)}}{{\left( {\frac{1}{{T_{1} blood post}} - \frac{1}{{T_{1 } blood pre}}} \right)}}$$

where *T*_1_ *myopost* and *T*_1_ *myopre* are the *T*_1_ values of post-contrast and native myocardium, and *T*_1_ *bloodpost* and *T*_1_ *bloodpre* are the *T*_1_ values of post-contrast and native blood, respectively.

### Myocardial biopsy: procedure and analysis

All aortic stenosis patients included underwent successful myocardial biopsy intraoperatively using the following standardised protocol: Once the chest was opened and cardiopulmonary bypass was established, a long near-transmural left ventricular “tru-cut” biopsy was taken. The aim was to undertake multiple biopsies provided that patient safety was not compromised. Upon completion of the biopsy, the myocardium was sutured, if required, and surgery continued as normal. The myocardial biopsies were immediately fixed in warm buffered 4% formalin. Histological analysis of fibrosis was undertaken on 3-µm-thick sections from formalin-fixed, paraffin-embedded endomyocardial biopsies using a special collagen stain (Picrosirius red). Quantification was performed using standardised semiautomatic image analysis software (Nikon Advanced Research NIS Elements imaging software, NIS elements AR 4.10.02, Nikon) on images of 14 randomly chosen consecutive high-power fields (×200 magnification), which were obtained with a Nikon Eclipse E400 light projection microscope (Nikon, Minato, Tokyo, Japan). The 14 high power fields equaled 1 mm^2^. Endocardium, subendocardial fibrosis and subendocardial fat, procedure-related optically empty spaces and epicardial fat were eliminated from analysis. Subsequent image analysis was performed to determine the level of fibrosis including reactive, band-like perimysial collagen depositions and perivascular fibrosis, defined as collagen surrounding arterioles. Fibrosis was calculated as the collagen volume fraction (%) per square millimeter, as previously described [19].

### Statistical analysis

The CMR native *T*_1_ values, partition coefficients, and ECVs were compared to the histologically identified collagen volume fraction (CVF). Intraclass correlation coefficients (ICC) were calculated using ‘R’ (R Foundation for Statistical Computing, Vienna, Austria) and used to assess agreement in the healthy volunteer ‘precision’ cohort. For the aortic stenosis ‘correlation with CVF' cohort, graph-plotting and analysis was again undertaken using the statistics package ‘R’. The six models, A–F, described for CMR estimation of myocardial diffuse fibrosis were compared to identify the most reproducible model (from the volunteer precision cohort) and most accurate/correlating with CVF (from the aortic stenosis biopsy cohort). There is no published method of incorporating both precision and accuracy (via correlation with CVF) in one single measurement for such imaging work. We therefore proposed and calculated the product of the scan-rescan *R* ^2^ values for the volunteer (precision) and ECV-histological fibrosis *R* ^2^ for aortic stenosis biopsy patients (correlation with CVF) as a single combined measure of correlation with CVF and precision for this imaging modality, described in this manuscript as Product of *R* ^2^.

## Results

### Reproducibility-precision: the volunteer cohort

**Fig. 3:**
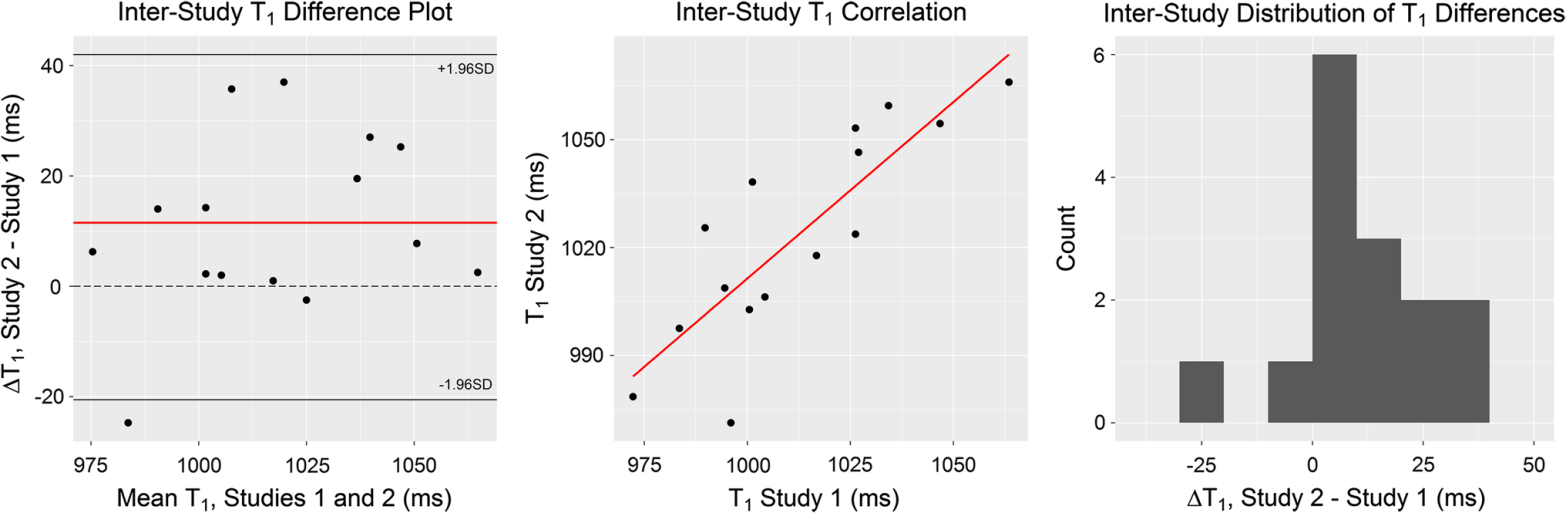
Bland–Altman analysis of *T*_1_, including mean difference, correlation, and distribution of differences plots. Mean difference = 11.5 ms (*red line* on difference plot) with limits of agreement at −21 and 43 ms; the line of zero difference (*dashed*) is within the limits of agreement

**Fig. 4:**
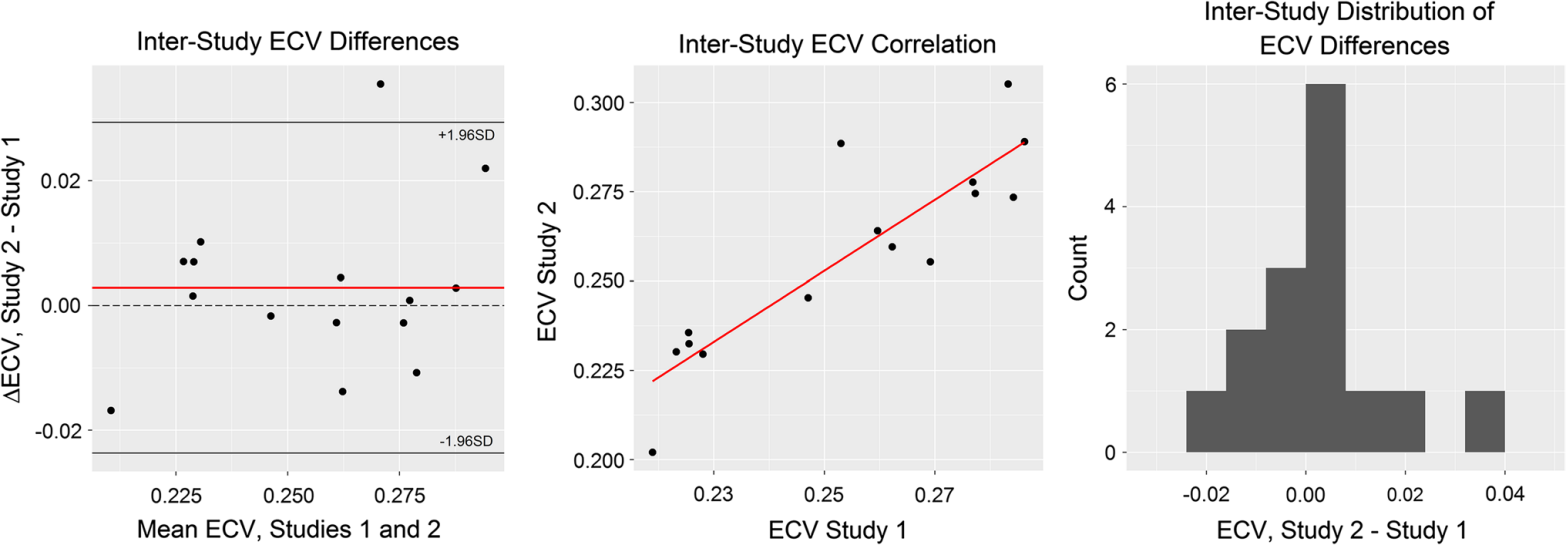
Bland–Altman analysis of ECV, including mean difference, correlation, and distribution of differences plots. Mean difference = 0.003 (*red line* on difference plot) with limits of agreement at −0.024 and 0.029; the line of zero difference (*dashed*) is within the limits of agreement

Fifteen healthy volunteers, eight males, mean age = 31 ± 5 years, were recruited and the reproducibility of their corresponding native *T*_1_ maps, post Gd *T*_1_ maps, partition coefficient and ECV results are shown in Table 2. All volunteers had an HR < 90 bpm for both scans, and; therefore, the higher resolution sequence was used. Participants tolerated both scans well with no complications and excellent *T*_1_ mapping images were obtained. No volunteer was excluded due to respiratory drift, cardiac motion artefact, or inappropriate cardiac triggering. There was no statistically significant difference between the two scans with regards to heart rate, blood pressure, or haematocrit (*p* = 0.27, *p* = 0.19, *p* = 0.89, respectively). Both interstudy *T*_1_ and interstudy ECV differences showed excellent results (Bland–Altman shown in Fig. 3 for native *T*_1_ and Fig. 4 for ECV, respectively).

**Table 2:** Showing interscan reproducibility

Interstudy reproducibility								
	Basal	Repeat	Mid	Repeat	Native *T*_1_ mapping	Post Gd mapping	Partition coefficient	ECV
					ICC *p* value	ICC *p* value	ICC *p* value	ICC *p* value
Model A			x		0.90 *P* < 0.001	0.72 *p* = 0.014	0.53 *p* = 0.098	0.84 *p* = 0.001
Model B	x				0.84 *p* = 0.001	0.68 *p* = 0.018	0.4 *p* = 0.17	0.75 *p* = 0.006
Model C			x	x	0.88 *p* < 0.001	0.82 *p* = 0.002	0.81 *p* = 0.002	0.94 *p* < 0.001
Model D	x	x			0.80 *p* = 0.001	0.75 *p* = 0.006	0.66 *p* = 0.024	0.88 *p* < 0.001
Model E	x		x		0.90 *p* < 0.001	0.71 *p* = 0.013	0.54 *p* = 0.08	0.88 *p* < 0.001
Model F	x	x	x	x	0.88 *p* < 0.001	0.79 *p* = 0.003	0.81 *p* = 0.002	0.93 *P* < 0.001

The six models A–F incorporating increasing levels and averaging of myocardial *T*_1_ maps are shown. Model C–F showed the best extracellular volume (ECV) reproducibility

*ICC* intraclass correlation

Both intra- and interobserver variability were excellent. Intraobserver ICCs were: 0.995 for native *T*_1_ (*p* < 0.001), 0.996 for partition coefficient (*p* < 0.001) and 0.999 for ECV (*p* < 0.001). Interobserver ICCs were: 0.993 for native *T*_1_ (*p* < 0.001), 0.985 for partition coefficient (*p* < 0.001), and 0.997 for ECV (*p* < 0.001).

### Correlation with collagen volume fraction: The aortic stenosis cohort

**Fig. 5:**
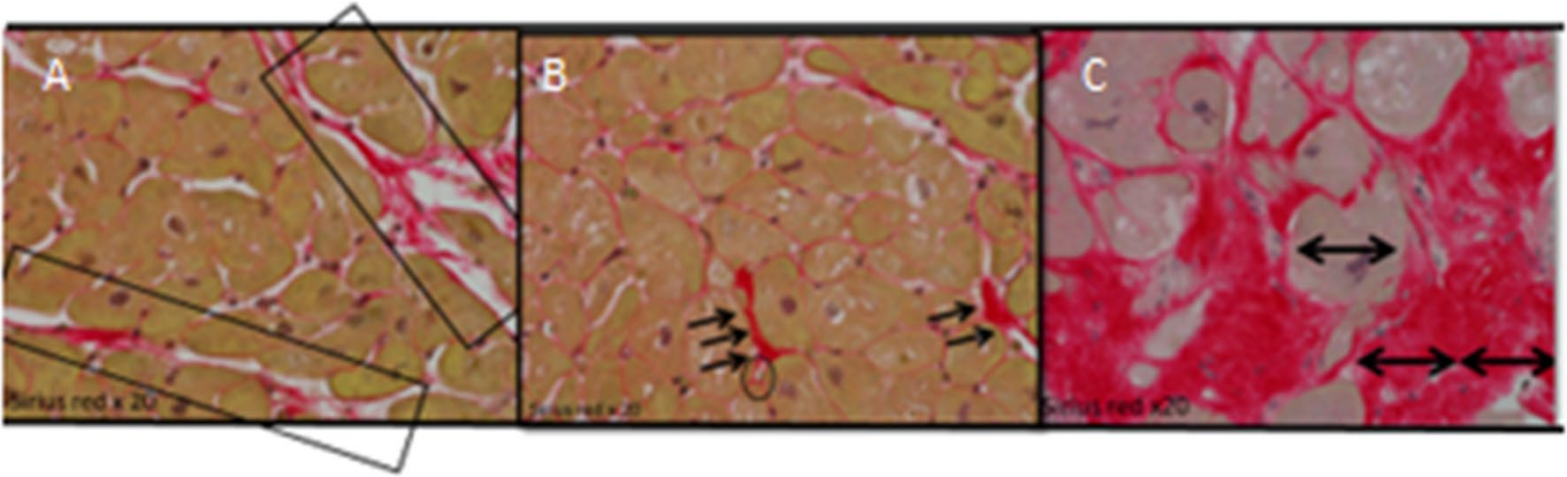
Samples from the intraoperative myocardial biopsies stained with Picrosirius red. **a** Shows fibrous septae, which show perivascular collagen to support mural arteries. There is no scarring, but only a slight increase in collagen fibres surrounding each cardiomyocyte (*light red*). **b** Shows interstitial fibrosis only, each cardiomyocyte is supported by a thin collagen layer (*light red*), there is only focal perivascular(pericapillary–capillary encircled) increase in collagen fibers (*dark red* area annotated). **c** Shows an annotated red area, qualifying as scar, as the dimension of the area exceeds double the diameter of the adjacent hypertrophic cardiomyocyte indicated with ⟷

Ten consecutive patients with symptomatic severe aortic stenosis scheduled for surgical valve replacement underwent CMR and intraoperative myocardial biopsies: eight male, mean age = 71 ± 10 years, and three with significant major epicardial coronary artery disease (defined as disease >50% of lumen diameter) requiring CABG at the time of the operation. Of these patients, five received treatment for hypertension and their blood pressure was well controlled at the time of the CMR (BP < 140/90 mmHg), four had treated hyperlipidaemia, two had chronic obstructive pulmonary disease requiring bronchodilators and two had prior distant history of neoplasia (breast/bladder). Of the three patients requiring CABG, one had 2-vessel CABG (left anterior descending (LAD) and circumflex) and the other two had single vessel CABG (one of the LAD and one of the intermediate coronary artery respectively). No patients suffered from diabetes. All patients tolerated the CMR well and the *T*_1_ mapping images were free from disqualifying artefacts and were diagnostic in all participants. In one patient the initial native *T*_1_ maps needed repeating in view of poor breathholding. This was appreciated during the scan, and therefore was repeated before proceeding to Gd administration. The repeat maps were of acceptable quality with no apparent respiratory drift, cardiac motion artefact, or inappropriate cardiac triggering. All patients had apical–lateral wall biopsies taken intraoperatively and no patients had biopsies from all three basal, mid, and apical levels due to surgical concerns. One patient had to be taken back to theatre within 24 h from surgery for bleeding, which may have been related to the apical myocardial biopsy sampling. The patient made a good recovery and discharged home within five days with no further sequela. No other side-effects relating to the myocardial biopsies were seen. Therefore, the basal and/or mid-myocardial *T*_1_ values were taken as a surrogate for a global *T*_1_ value and were correlated with histology from the apical–lateral wall. Microscopic views of the sections prepared from the biopsies are shown in Fig. 5.

**Fig. 6:**
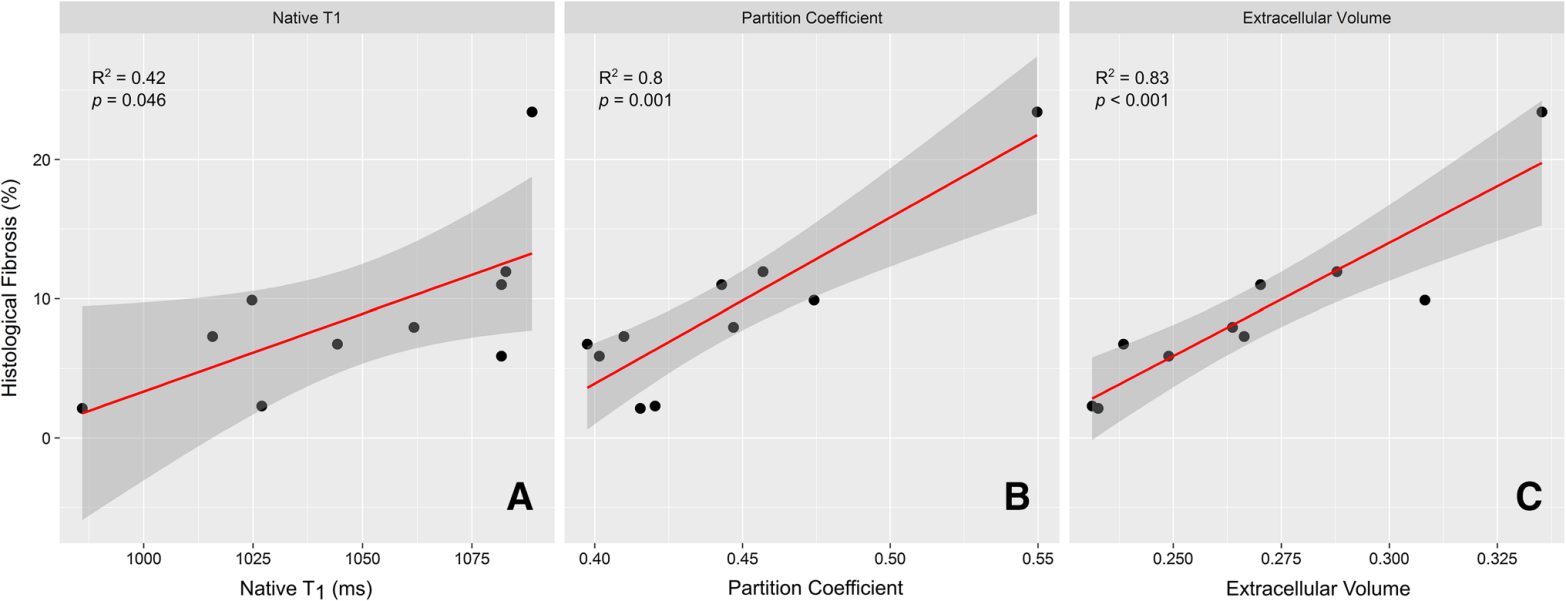
The agreement comparing (apical) histological CVF against ECV (**a**), native *T*_1_ mapping (**b**), and partition coefficient (**c**). There was good agreement between CVF and all the imaging parameters; however, partition coefficient and ECV performed considerably better than native *T*_1_ mapping alone

The relative correlation with CVF of native *T*_1_ maps, partition coefficient, and ECV were assessed using histology. The histologically derived CVF was plotted against each of these parameters, as shown in Table 3. Native *T*_1_ values from model F (which had shown best correlation) showed a moderate, statistically-significant correlation with the histologically obtained fibrosis burden, with *R* ^2^ = 0.42, and *p* = 0.046. However, the partition coefficient and ECV both showed strong correlations with histology, with *R* ^2^ = 0.81 and *R* ^2^ = 0.83, and these both were statistically significant, with *p* < 0.001 (Table 3; Fig. 6).

**Table 3:** showing the correlation of extracellular volume (ECV) by each model with histological collagen volume fraction (CVF) from apical–lateral histology

Correlation of ECV with histological fibrosis							
	Basal level	Basal repeated	Mid-level	Mid-level repeated	ECV (%)	*R* ^2^ with histology	*p* value
Model A			x		27.47	0.36	0.068
Model B	x				26.67	0.77	0.001
Model C			x	x	26.86	0.49	0.025
Model D	x	x			26.79	0.81	<0.001
Model E	x		x		27.07	0.72	0.002
Model F	x	x	x	x	26.83	0.83	<0.001

It can be argued that in view of other myocardial pathologies co-existing with aortic stenosis such as oedema or ischaemia, CVF might not be the gold standard for fibrosis comparison. Therefore, this table should be interpreted in the appropriate clinical context. In patients with aortic stenosis, it would appear that ECV derived from the basal slice correlated better with CVF than the mid-ventricular level, but a combination of both the basal and mid had the best correlation. These results would suggest that further research is warranted to show whether imaging of basal and mid-ventricular level should be routinely undertaken in patients with aortic stenosis

**Fig. 7:**
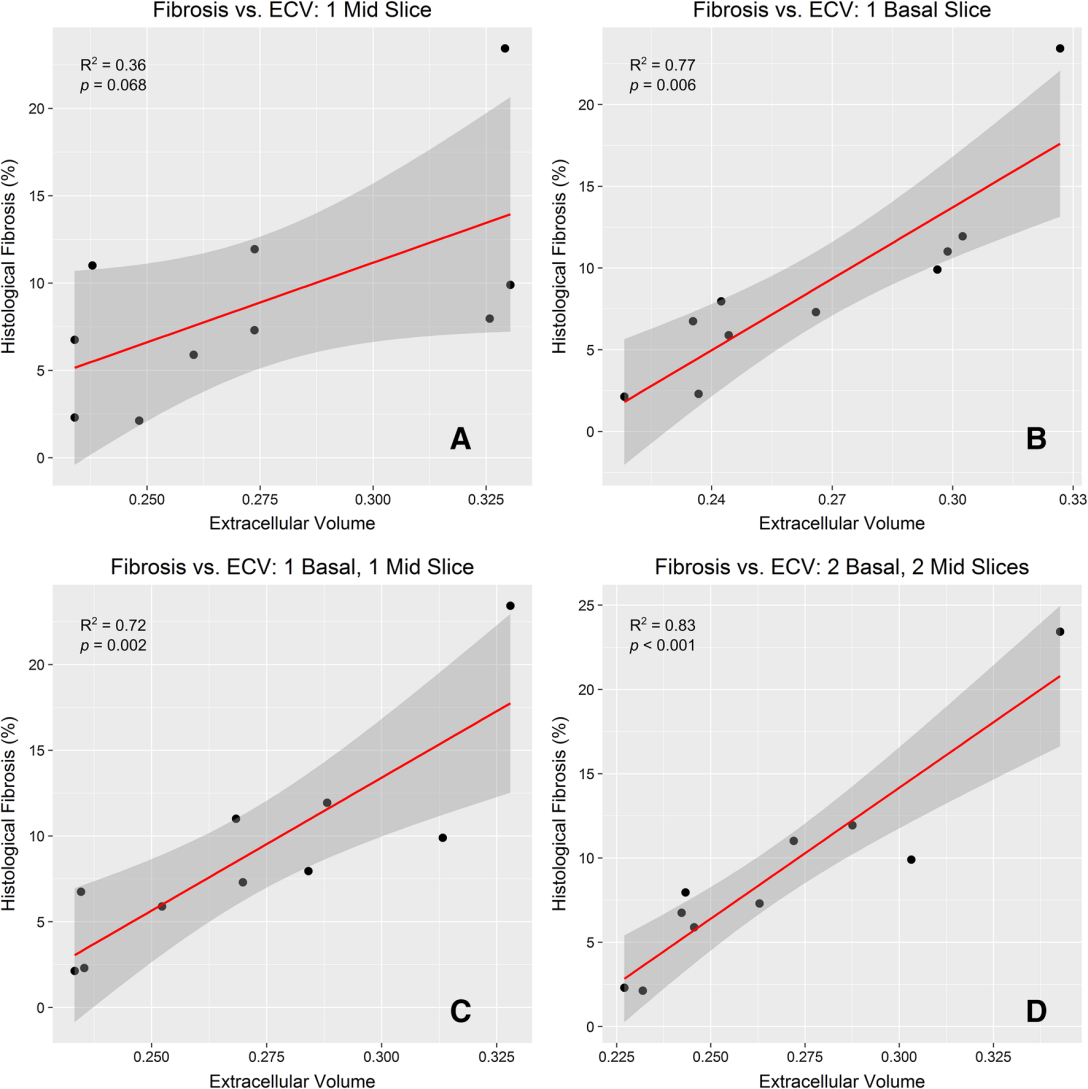
**a** Showing the correlation of (apical–lateral) histological collagen volume fractions (CVF) with ECV calculated from CMR at a single mid-ventricular level, corresponding to Model A and showing only mild non-significant correlation. **b** Showing correlation of histological fibrosis with a single basal level (Model B), showing that for this pathology the correlation increases and has now become significant. **c** Showing correlation between the average of one mid-level and one basal level (Model E) with histology showing a significant correlation. **d** Represents Model F with two basal and two mid-slices showing that this model demonstrated the strongest correlation (*R* ^2^ = 0.83, *p* < 0.001). This work confirms that including incrementally more levels imaged improves accuracy

Having established that ECV was the most accurate imaging parameter from *T*_1_ mapping, we further investigated the incremental addition of more myocardial regions and averaged slices. When only one mid-level *T*_1_-map was used (i.e., Model A), the correlation with histology was only modest, and was not significant (*R* ^2^ = 0.36, *p* = 0.068), similar to data from published studies that used only one mid-level *T*_1_-map [14, 20]. Increasing the number of *T*_1_ maps, and including the basal level as well as the mid-ventricular-level in this averaging improved the correlation significantly: *R* ^2^ = 0.72, *p* = 0.002 when the average of a single basal and single mid-level image was used for ECV (Model E) and importantly *R* ^2^ = 0.83, *p* < 0.001 when acquisition was repeated at both basal and mid-levels (Model F) as shown in Table 3 and Fig. 7.

### Combining precision and correlation with collagen volume fraction

To allow us to clarify the relative incremental addition of imaging for each model combining both accuracy (via correlation with CVF) and precision we sought a single parameter for each model to incorporate both correlation with CVF and precision. However, such a single measure has not been described for *T*_1_ mapping. We therefore devised a new model, incorporating the product of the *R* ^2^ from the precision cohort and *R* ^2^ from the ‘correlation with CVF' cohort as a unique parameter for each model. The results are shown in Table 4, and indicate that the inclusion of a repeated basal image and a repeated mid-image, giving a total of four native and four post Gd *T*_1_ maps to calculate ECV, was the most precise method with the best correlation with CVF.

**Table 4:** Combining accuracy and precision in a single measurement, the Product of *R* ^2^ indicating that Model F offers the best imaging protocol (basal and repeat, mid-ventricular level and repeat) for optimal accuracy and precision

	Basal	Repeat	Mid	Repeat	Volunteer interscan *R* ^2^	ECV vs histology *R* ^2^	Product of *R* ^2^
Model A			x		0.759	0.356	0.27
Model B	x				0.722	0.773	0.56
Model C			x	x	0.808	0.486	0.39
Model D	x	x			0.637	0.809	0.52
Model E	x		x		0.623	0.715	0.45
Model F	x	x	x	x	0.778	0.829	0.65

*ECV* extracellular volume

## Discussion

Any new CMR sequence needs to show both good accuracy and precision to become clinically relevant. In this work, we used two cohorts to establish precision and correlation with CVF (as a surrogate for accuracy) for an as yet non-validated *T*_1_ mapping sequence. Furthermore, this is the first time a study has reported a model of incremental image acquisition and averaging of basal and mid-ventricular *T*_1_-measurements as a means of obtaining an average of the global *T*_1_-mapping ECV. The volunteer cohort demonstrated that four models of slice acquisition and averaging showed an excellent interscan correlation. Model A native *T*_1_ mapping (one native *T*_1_ mid-ventricular level slice), Model E native *T*_1_ mapping (average of one basal and one mid-level slice native *T*_1_), Model C ECV (average of ECV of two mid-level slices), and Model F ECV (average ECV of two basal and two mid-slices) all showed excellent interscan reproducibility, with *R* ^2^ ≥ 0.90.

The correlation with CVF was assessed using a cohort of ten patients with aortic stenosis scheduled for surgical valve replacement who had CMR using our model of incremental addition of imaging acquisitions and intraoperative biopsies. This confirmed that in aortic stenosis patients, measuring ECV in the mid-ventricular wall alone (Model A) showed only a trend towards weak correlation with histological collagen volume fraction (CVF), *R* ^2^ = 0.36, *p* = 0.068. A model utilising the basal level alone (Model B) showed good correlation with histology (*R* ^2^ = 0.77, *p* = 0.001), while a model based on one basal level being imaged twice and averaged (Model D) showed better correlation (*R* ^2^ = 0.81, *p* < 0.001). The best correlation was achieved by a model having one basal and one mid-ventricular slice being imaged twice each and averaged (Model F, *R* ^2^ = 0.83, *p* < 0.001). Incorporating both precision and correlation with CVF parameters into the Product *R* ^2^, showed that ECV calculated from Model F was the most accurate and precise. We speculate that the improved correlation and precision associated with Model F may relate to the incorporation of further areas of the myocardium (both basal and mid-ventricular level) and the repeat scan may serve to average out any potential errors associated with imaging a single slice.

This work therefore supports the following conclusions: firstly, the 11 heart beat MOLLI prototype incorporating native 5b(3b)3b and post-contrast 4b(1b)3b(1b)2b schemes, was well tolerated by volunteers and patients. Secondly, when isolated native *T*_1_ mapping was used (without post-contrast *T*_1_ maps), it is reproducible in healthy volunteers, especially when Model A (one mid-slice), Model C (one mid-slice repeated), Model E (one basal and one mid-slice), or Model F (one basal and one mid-ventricular level, both repeated) are used. Repeating native *T*_1_ maps does not appear to offer additional improvement in precision, as Model A performed at least as well as Models C, E and F. Thirdly, both partition coefficient and ECV have been shown to be more accurate than native *T*_1_ maps alone. Therefore, we propose that post-Gd imaging (with or without haematocrit sampling) should be routinely undertaken when looking for an estimation of diffuse fibrosis in patients. Fourthly, a single mid-ventricular ECV measurement appears to show poorer correlation with CVF in patients with aortic stenosis (as shown by Model A). Providing additional image repeats, such as model C, E or F for example, appear to show improved correlation with CVF. Although our study is not without its limitations, particularly regarding the use of the CVF as the reference standard, further work could consider whether the additional improvement in correlation provided by models C, E, and F, for example, might be of clinical importance. Finally, the most accurate and precise model is Model F, whereby imaging of one basal and one mid-ventricular level slice, with both being repeated once, is performed both in the native state and after Gd administration.

We therefore recommend that this should be considered if there is available scanner time and the patients can tolerate it. However, increasing scanner time (even by 5–7 min) might not be possible in many busy CMR units. As such, undertaking repeats of basal and mid-ventricular levels might not be an option. Nonetheless, even in busy units it should be possible to undertake repeat imaging of a single level, which will only add two breathholds to the scan time. Therefore, if a unit undertakes a mid-ventricular slice *T*_1_ mapping routinely in the native and post-contrast state, we recommend that imaging at the same level is repeated once. This will provide additional improvement in the correlation-precision, but also safeguard against potential poor breathholding, poor coregistration, or motion correction in one of the two acquisitions. Such a practical compromise will only increase scanner time by about 30 s. It should be noted that this relation only holds true for the 11HB sequence we have tested, and other MOLLI sequences might not behave in a similar way.

This study has certain limitations. Firstly, our samples for both cohorts were small; however, this is in line with other recently published studies, which also use small (fewer than ten patients) datasets for histological validation [21, 22]. Secondly, we used healthy volunteers for the interscan reproducibility, as this was practically easier to achieve. As aortic stenosis patients can be more breathless and might have difficulties with breathholding, it is possible that the reproducibility might not be as good as for healthy controls. However, recently published work [23, 24] has shown that reproducibility in aortic stenosis was excellent, and therefore, we would not have anticipated different results to our controls, as *T*_1_ mapping in our aortic stenosis patients was of very good quality. Furthermore, we elected not to include apical *T*_1_ mapping in any of our *T*_1_ mapping strategies, as this region is known to suffer increased partial-volume artefact at the endocardial and epicardial borders, which are not perpendicular to the image slice in the apical short-axis region [25]. Moreover, we did not undertake intra-study reproducibility which could have provided additional information. We felt that intrascan reproducibility for ECV would be difficult to interpret as by default the post-Gd *T*_1_ maps would be taken at different times. In addition, our myocardial biopsies were taken from the apico-lateral wall. Nonetheless, earlier studies have shown that, unlike replacement fibrosis, where there is a significant difference between basal and apical areas [26], interstitial fibrosis tends to follow a more homogenous pattern [23, 27] in patients with aortic stenosis. Therefore, we feel that our results are valid for the apico-lateral wall biopsies to represent global diffuse fibrosis measurement in these patients. Finally, it is appreciated that other pathological processes might affect *T*_1_/ECV in aortic stenosis including oedema [25] and ischaemia [28, 29]; therefore, the correlation between *T*_1_/ECV and CVF should be regarded as an association rather than causative, as there could be multiple aetiologies that might increase *T*_1_/ECV.

## Conclusion

This work was the first to utilise *T*_1_ mapping strategies with increasingly inclusive additional imaging for averaging the septal ECV measurements, going from one slice imaged to a total of four images (one basal repeated and one mid repeated) to improve both the correlation with CVF and the precision of the *T*_1_-measurement (both before and after Gd), comparing histological CVF estimation of fibrosis to the *T*_1_-mapping outcomes of native *T*_1_ maps, partition coefficient and ECV. We have shown that the 11 heartbeat MOLLI sequence used in this research, native 5b(3b)3b with post-contrast 4b(1b)3b(1b)2b, was well-tolerated in both volunteers and patients with aortic stenosis. Furthermore, it was both accurate and precise, particularly as incremental imaging of additional basal and mid-ventricular slice images are included. Finally, this work also confirms that partition coefficient and ECV can be accurately used to correlate with histological CVF estimation of diffuse fibrosis.
